# Muscle contraction phenotypic analysis enabled by optogenetics reveals functional relationships of sarcomere components in *Caenorhabditis elegans*

**DOI:** 10.1038/srep19900

**Published:** 2016-01-29

**Authors:** Hyundoo Hwang, Dawn E. Barnes, Yohei Matsunaga, Guy M. Benian, Shoichiro Ono, Hang Lu

**Affiliations:** 1 School of Chemical & Biomolecular Engineering, Georgia Institute of Technology, Atlanta, GA, USA; 2 Department of Pathology, Emory University, Atlanta, GA, USA; 3 Interdisciplinary Program of Bioengineering, Georgia Institute of Technology, Atlanta, GA, USA; 4 The Petit Institute for Bioengineering and Biosciences, Georgia Institute of Technology, Atlanta, GA, USA

## Abstract

The sarcomere, the fundamental unit of muscle contraction, is a highly-ordered complex of hundreds of proteins. Despite decades of genetics work, the functional relationships and the roles of those sarcomeric proteins in animal behaviors remain unclear. In this paper, we demonstrate that optogenetic activation of the motor neurons that induce muscle contraction can facilitate quantitative studies of muscle kinetics in *C. elegans*. To increase the throughput of the study, we trapped multiple worms in parallel in a microfluidic device and illuminated for photoactivation of channelrhodopsin-2 to induce contractions in body wall muscles. Using image processing, the change in body size was quantified over time. A total of five parameters including rate constants for contraction and relaxation were extracted from the optogenetic assay as descriptors of sarcomere functions. To potentially relate the genes encoding the sarcomeric proteins functionally, a hierarchical clustering analysis was conducted on the basis of those parameters. Because it assesses physiological output different from conventional assays, this method provides a complement to the phenotypic analysis of *C. elegans* muscle mutants currently performed in many labs; the clusters may provide new insights and drive new hypotheses for functional relationships among the many sarcomere components.

Contractile activity of muscle is required for animal movement. The sarcomere, the fundamental unit of muscle contraction, is composed of hundreds of proteins and highly conserved in most animals. The body wall muscle of the nematode *Caenorhabditis elegans* (*C. elegans*) is a valuable model system to study the assembly, maintenance, and function of the sarcomere in striated muscle. Many genes that are required for organization of sarcomeres and the regulation of muscle contraction have been identified in *C. elegans*. The proteins encoded by these genes have been localized in the sarcomere, and their molecular and/or genetic interactions have been partly identified. For instance, *unc-54* encodes myosin heavy chain B which is a component of the thick filaments^1^,^2^,^3^. *unc-22* encodes a large muscle filament protein, twitchin, that is localized to the A-bands, where the thick filaments and the thin filaments are overlapped^4^,^5^. *unc-89* encodes a homologue of the vertebrate protein, obscurin, a structural component of the M-line, where the thick filaments are anchored^6^. UNC-98 and UNC-96 are also mainly localized to the M-lines and are required for organization of the thick filaments^7^,^8^,^9^. *unc-27* and *lev-11* encode troponin I and tropomyosin, respectively, which are parts of the thin-filament associated regulatory system for actin-myosin interactions^10^,^11^. *unc-60B* and *unc-78* encode a muscle-specific actin depolymerizing factor/cofilin isoform and a homologue of actin-interacting protein 1, respectively, which promote actin dynamics and are required for proper actin filament assembly^12^,^13^. In 2011, it was conservatively estimated that at least 200 proteins are required for the assembly, maintenance and function of the sarcomere in *C. elegans* body wall striated muscle^14^. We continue to identify new components of the nematode sarcomere, and the sarcomere in general. However, the biochemical and physiological functions of these proteins are only partly understood. A major challenge is to understand how these proteins work together to build, maintain and regulate the sarcomere.

A very important contribution of the *C. elegans* model system has been, and continues to be, the genetic analysis of sarcomere components – “classical” forward genetics, reverse genetics, transgenesis, and more recently the prospect of gene editing. In fact, classical genetics led to the first identification of many of these conserved sarcomeric proteins in any system; examples include twitchin, UNC-89 (obscurin), UNC-112 (kindlin), UNC-45 (myosin chaperone), and UNC-78 (AIP1). However, phenotypic analysis of these mutants, especially at the physiological level has been limited. This is partially because the phenotyping so far has mainly relied on subjective characterization, and the descriptors have been fairly broad. This is evident in the naming of many muscle-affecting mutants and genes: there are many “unc”s (uncoordinated) and this broad descriptor includes mutants that show a variety of abnormal body movements. An often-used technique is to simply count the number of times a worm bends in C-shaped fashion in liquid over a specified time, usually one minute^15^. However, the locomotion of nematodes in liquid or on semi-solid surfaces like agar is different, and it is likely that a worm uses different gait and different energy expenditure during swimming and crawling locomotion. Recently there have been a few studies in which the swimming or crawling locomotion of *C. elegans* was investigated through computer vision-based image analysis. For instance, Nahabedian *et al.* quantitatively measured the maximum bending amplitude of crawling worms which have defects in genes encoding muscle focal adhesion components^16^. Krajacic *et al.* quantified various biomechanical properties such as bending frequency and amplitude of swimming in synaptic mutants^17^. These methods allow more nuanced phenotyping, low user bias, and therefore potentially can extract more information; furthermore, it is possible that assays that measure other attributes of muscle contraction and relaxation may expand the repertoire of descriptors of sarcomere functions.

The ability to mechanically analyze individual muscle cells (aka fibers) from vertebrate skeletal muscle, has a long history and has yielded many insights into muscle physiology^18^. It is even possible to conduct these types of experiments using various muscle cells (e.g. thoracic, indirect flight and jump muscles) from *Drosophila melanogaster*^19^, in which the influence of mutations in various sarcomeric proteins can be determined. Unfortunately, due to the small size of *C. elegans* and its mono-nucleated muscle cells, these single muscle fiber experiments cannot be done. Therefore, we have taken an alternative approach to measuring the kinetics of muscle contraction/relaxation. To control the contractile activity of *C. elegans* muscle, we utilized optogenetics, a technique that permits control of the electrical activities of neurons with visible light. *C. elegans* moves by the alternating contraction and relaxation of dorsal and ventral body wall striated muscle cells. We employed a worm strain carrying *unc-17p::ChR2*(*H134R*)*::YFP*, which expresses the light-induced channelrhodopsin-2 (ChR2) in its cholinergic motor neurons, to control the contractile activity of the body wall muscle cells and hence the locomotion of the whole nematode, as first described by Liewald *et al.*^20^. When exposed to a blue light (450–490 nm), channel rhodopsin promotes the uptake of cations, consequently activating the motor neurons and inducing muscle contraction and shrinkage of the entire nematode body. When the light is turned off, motor neurons are no longer activated, muscle relaxes, and the nematode body relaxes to its normal size. Quantitative parameters, including rate constants for contraction and relaxation, could be extracted from these optogenetic experiments. This was performed on wild type nematodes and loss of function mutants for 15 genes that encode proteins of the sarcomere. In this work, we also use clustering analysis based on those parameters to look for information on the functional relationships of the sarcomere components. Our approach should provide a complement to existing behavioral assays for the analysis of *C. elegans* muscle mutants.

## Results

Quantitative study of muscle contraction and relaxation kinetics in *C. elegans* was enabled by optogenetically-induced activation of motor neurons that induce muscle contraction. To investigate roles of sarcomeric proteins in the behavior of nematodes, mutants in 15 sarcomeric proteins were examined (Table 1). This set of mutants sample mutations in genes encoding proteins localized to all major structures of the sarcomere (A-bands, I-bands, thick filaments, thin filaments, M-lines and dense bodies (Z-disk analogs in nematode muscle)). To minimize the influence of gross defects in sarcomere structure, our collection of mutants were biased towards those genes whose null phenotypes have mild to no defects in sarcomere structure (*mak-1*, *atn-1*, *scpl-1*, *lim-9, unc-27, unc-78*), or alleles were chosen that exhibit mild or the mildest defects among existing alleles (*unc-22*(*e105*), *unc-54*(*s74*), *unc-60B* (*r398*)*, lev-11*(*x12*)). Many of our mutants were chosen that have minimal known defects on nematode locomotion (*unc-22*(*e105*), *mak-1*, *atn-1*, *scpl-1*, *dim-1*, *uig-1*, *lim-9*). This criteria was used so that we might determine if optogenetic assays might reveal more subtle defects in muscle function. Finally, our collection includes mutants in several known (UNC-27 (troponin I), LEV-11 (tropomyosin)) or suspected proteins (UNC-22 (twitchin)) that regulate muscle contraction.

**Figure 1:**
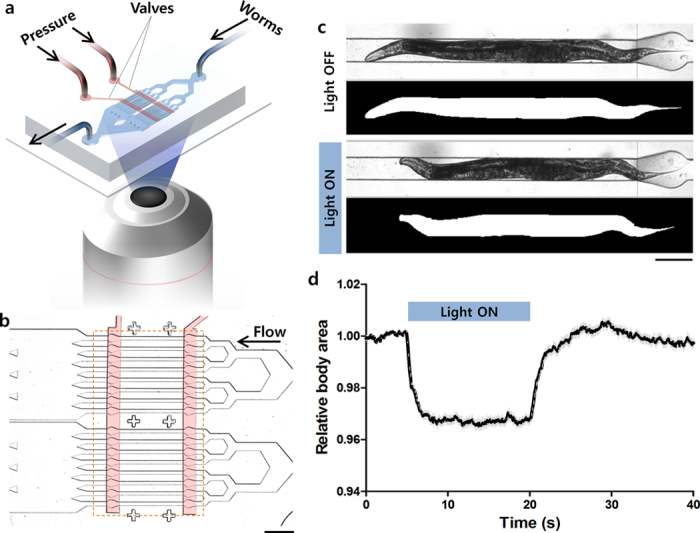
Optogenetic analysis of muscle contraction and relaxation kinetics in* C. elegans.* **(a)** Schematic diagram for the behavioral phenotypic analysis enabled by optogenetics, microfluidics, and image processing technologies. **(b)** A microfluidic device having sixteen parallel microchannels for simultaneous illumination and analysis of multiple animals trapped by two pneumatically-controlled microvalves (red). Scale bar = 500 μm. **(c)** Microscopic and segmented images of a wild-type animal trapped in the microchannel with (top) and without (bottom) the illumination of blue light. Scale bar = 100 μm. **(d)** Temporal change in the body size of the wild type animals that was calculated from the segmented images and normalized by average value in the first 5 s. The light was turned on for 15 s at the 5-s time point. Data represent mean ± s.e.m. *n* = 88.

To perform the optogenetic muscle contraction experiments, these sarcomere mutants were crossed into *zxIs6* [*unc-17p::ChR2*(*H134R*)*::YFP* + *lin-15*(+)] transgenic worms, which express ChR2 molecules in cholinergic motor neurons by the *unc-17* promotor^20^,^21^. In this study, we refined and applied a microfluidic device where multiple worms can be trapped in multiple channels and illuminated for ChR2 photoactivation simultaneously, previously designed for high-throughput studies of synaptic transmission^22^. The two-layer device has 16 parallel microchannels in the bottom layer, and two pneumatically-controlled valves in the top layer to open or close the channels (Fig. 1a,b). When the worms were loaded into the device and trapped in the microchannels, a blue light (450–490 nm) was illuminated. With the illumination of blue light, the cholinergic motor neurons in the nematodes were activated and their body muscles were contracted, resulting in decreased body size (Fig. 1c). Based on image processing for segmentation of worm bodies from the recorded images (Fig. 1c), we quantitatively tracked the change in the projected body area according to the light-induced muscle contraction (Fig. 1d). This method allowed us to perform high-throughput non-biased analysis of the kinetics of body wall muscle contraction and relaxation in *C. elegans*. Here the exposure time was set as 15 s based on preliminary experiments with various exposure times from 5 to 30 s; the contracted body area did not significantly change after 15 s of illumination. When the light was turned off, the relative body area began to increase due to relaxation of the body muscles (Fig. 1d). Wild type animals showed ~3% decrease in their projected body size and completed the contraction or the relaxation processes within around 5 s after turning on or off the light, respectively (Fig. 1d).

**Figure 2:**
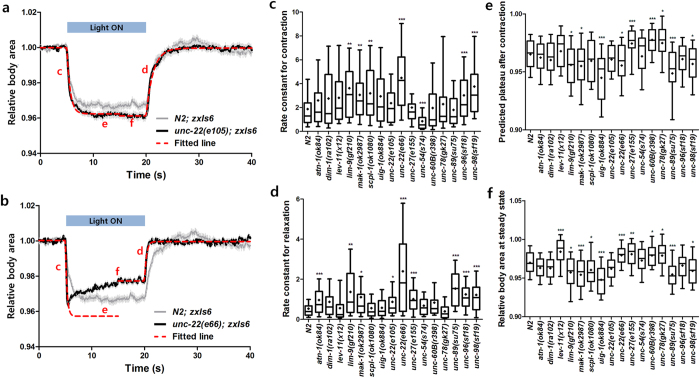
Quantitative analysis of the dynamic curves obtained from the optogenetic muscle contraction and relaxation assays in microfluidic devices. Graphs showing the optogenetic muscle contraction and relaxation processes of (**a**) *unc-22*(*e105*) and (**b**) *unc-22*(*e66*) mutants in comparison with that of the wild-type animals. The light was turned on for 15 s at the 5-s time point. Data represent mean ± s.e.m. All the plots obtained from the experiments with sixteen mutant strains were devided into two parts – contraction (0–15 s) and relaxation (15–40 s) processes–and fitted with one-phase decay and association models, respectively, to extract four quantitative parameters to describe the dynamic curves: (**c**) rate constant for contraction; (**d**) rate constant for relaxation; (**e**) plateau after contraction; and (**f**) relative body area at steady state. *n* ≥ 40. ^*^P < 0.01; ^**^P < 0.001; ^***^P < 0.0001.

The optogenetic assays for muscle contraction kinetics were conducted for all of the sarcomere mutant animals we have at hand (Supplementary Fig. 1). By fitting the dynamic curves of individual animals with a one-phase decay or association model and calculating rate constants (Fig. 2a,b), we quantitatively analyzed how fast they contract or relax the body wall muscles. Some muscle mutants including *lim-9*(*gf210*), *mak-1*(*ok2987*), *scpl-1*(*ok1080*), *unc-22*(*e66*), *unc-96*(*sf18*), and *unc-98*(*sf19*) contracted significantly faster than wild type animals, while *unc-54*(*s74*) showed significantly slower contraction compared to the wild type animals (*P* < 0.001; Fig. 2c). Mutant animals including *atn-1*(*ok84*), *lim-9*(*gf210*), *mak-1*(*ok2987*), *unc-22*(*e105*), *unc-22*(*e66*), *unc-27*(*e155*), *unc-89*(*su75*), *unc-96*(*sf18*), and *unc-98*(*sf19*) relaxed significantly faster than the wild type animals after turning off the illumination (*P* < 0.01; Fig. 2d). Interestingly, no mutants showed relaxation rates slower than wild type animals. Although the rate constants for contraction or relaxation provide valuable information, they do not fully describe the range of behaviors observed among the mutants. For instance, *unc-22*(*e66*) showed a unique behavior in that the body size gradually increased due to a slight relaxation of the body muscles, just after the maximum contraction point where the relative body size had a minimum value, even though they were still illuminated (Fig. 2b). Interestingly, a different allele of the same gene showed very different behavior: the relative body area of *unc-22*(*e105*) at the maximum contraction was similar to that at the steady state, similar to wild type (Fig. 2a). Such characteristics of the body area change due to the muscle contraction is not captured by the rate constants for contraction and relaxation. Therefore, we extracted two more features from the fitted curves: predicted plateau after contraction, which indicates the relative body area predicted at infinite times after the contraction process (“e” in Fig. 2a,b); and relative body area at steady state, which is the average relative body area during 5 s before the relaxation process (“f” in Fig. 2a,b). Some mutant animals including *lim-9*(*gf210*), *mak-1*(*ok2987*), *uig-1*(*ok884*), *unc-22*(*e66*), *unc-89*(*su75*), and *unc-98*(*sf19*) showed significantly smaller predicted plateau after contraction than the wild type animals, while *unc-27*(*e155*), *unc-60B*(*r398*), and *unc-78*(*ok27*) showed significantly higher values (*P* < 0.01; Fig. 2e). The relative body area of some mutant animals including *lim-9*(*gf210*), *mak-1*(*ok2987*), *scpl-1*(*ok1080*), *uig-1*(*ok884*), *unc-89*(*su75*), and *unc-98*(*sf19*) was significantly smaller than that of the wild type animals at the steady state, while that of *lev-11*(*x12*), *unc-22*(*e66*), *unc-27*(*e155*), *unc-60B*(*r398*), and *unc-78*(*gk27*) was significantly larger than the wild type (*P* < 0.01; Fig. 2f).

The difference between the plateau after contraction and the relative body area at steady state indicates the ability to maintain the contraction of the muscles. No significant difference between those two values means the muscle contraction is maintained. Amongst all mutants examined, *lev-11*(*x12*) and *unc-22*(*e66*) show smaller or similar plateau after contraction than the wild type animals, while their relative body sizes at the steady state are significantly larger than that of wild type. In these mutants, the muscles relax after the contraction process despite the sustained light illumination. Interestingly, both *lev-11*(*x12*) and *unc-22*(*e66*) also show twitching locomotion while freely moving. This suggests that the inability to maintain the contraction of muscles might be related to the twitching motility observed in free locomotion.

**Figure 3:**
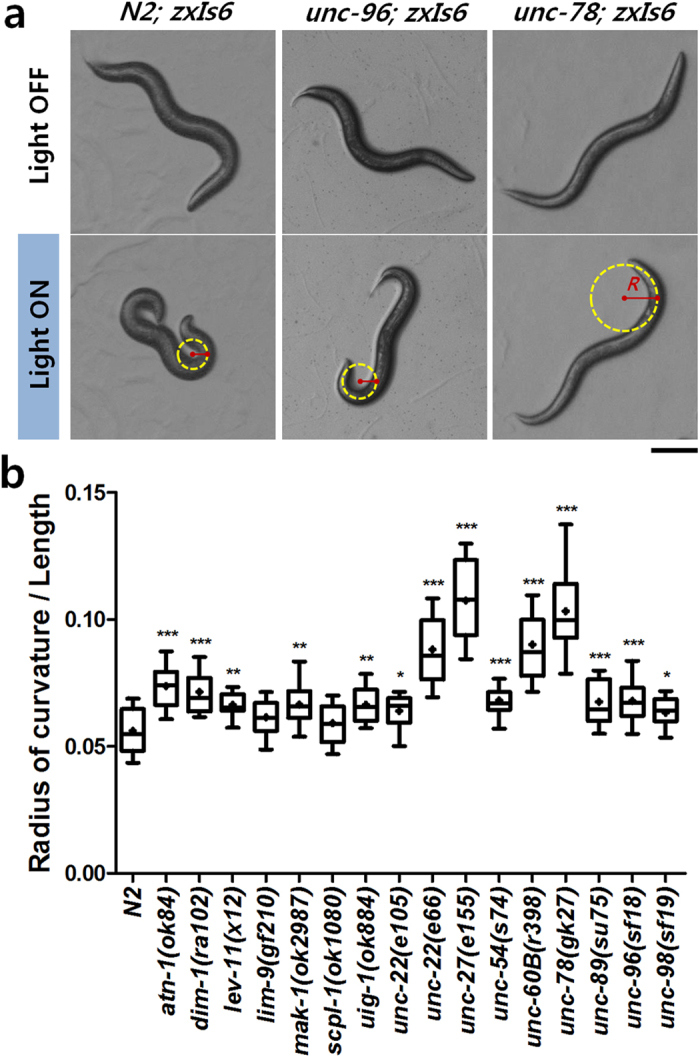
Quantitative analysis of the minimum radius of curvature at the full contraction of body wall muscles in *C. elegans* sarcomere mutants on plates. (**a**) Representative images showing the body postures of the wild-type, *unc-96*(*sf18*), and *unc-78*(*gk27*)) without (top) and with (bottom) the illlumination of blue light to induce the full contraction of their body wall muscles. Scale bar = 100 μm. (**b**) The minimum radius of curvature of the wild-type and sixteen sarcomere mutant strains at full muscle contraction and normalized by the length of the animals. *n* ≥ 25. ^*^P < 0.01; ^**^P < 0.001; ^***^P < 0.0001.

When nematodes carrying ChR2 in their cholinergic motor neurons were illuminated with blue light on an agar surface, they tended to coil up their bodies due to the contraction of their body muscles (Fig. 3a). We expected that loss of function mutations in sarcomeric proteins might induce distinctive phenotypic defects not only in contraction and relaxation kinetics, but also in this coiling behavior. Thus, we also measured the minimum radius of curvature when their contracted muscles had achieved this coiled body posture. It is likely that the minimum radius of curvature as being inversely proportional to a maximum “ability” of the body to bend. All of the muscle mutants, except *scpl-1*(*ok1080*) and *lim-9*(*gf120*), showed significantly larger minimum radii of curvature (*P* < 0.01; Fig. 3b).

**Figure 4:**
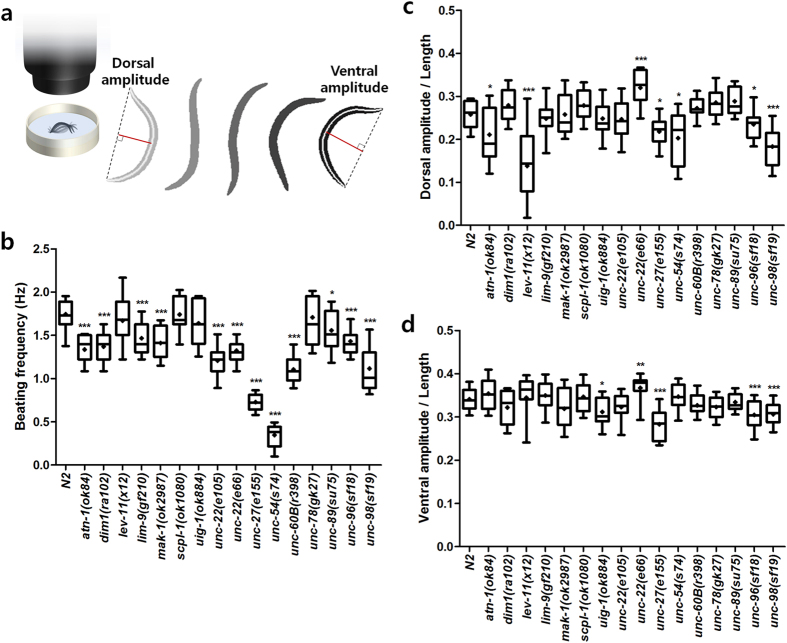
Swimming locomotion analysis of *C. elegans* sarcomere mutants. **(a)** Schematic diagram of swimming locomotion assays. The nematodes were put into M9 buffer solution and their swimming locomotion was recorded. Three quantitative parameters were extracted from the threshold-segmented images: **(b)** beating frequency, and **(c)** doral and **(d)** ventral bending amplitudes normalized by the length of the animals. *n* ≥ 20. ^*^P < 0.01; ^**^P < 0.001; ^***^P < 0.0001.

For comparison, we also conducted the conventional assays of swimming and crawling locomotion. For the analysis of swimming behavior, the nematodes including wild type and the muscle mutants were put into a plate containing M9 buffer and their swimming motion was recorded (Fig. 4a). By post-processing the segmented images, three parameters were measured – beating frequency, dorsal bending amplitude, and ventral bending amplitude. Most of the muscle mutant animals including *atn-1*(*ok84*), *dim-1*(*ra102*), *lim-9*(*gf210*), *mak-1*(*ok2987*), *unc-22*(*e105*), *unc-22*(*e66*), *unc-27*(*e155*), *unc-54*(*s74*), *unc-60B*(*r398*), *unc-89*(*su75*), *unc-96*(*sf18*), and *unc-98*(*sf19*) beat significantly slower than the wild type (*P* < 0.01; Fig. 4b). Some mutant animals including *atn-1*(*ok84*), *lev-11*(*x12*), *unc-27*(*e155*), *unc-54*(*s74*), *unc-96*(*sf18*), and *unc-98*(*sf19*) bent their bodies less than in dorsal direction than the wild type (*P* < 0.01; Fig. 4c). Few mutant animals including *uig-1*(*ok884*), *unc-27*(*e155*), *unc-96*(*sf18*), and *unc-98*(*sf19*) showed smaller amplitudes in ventral bending compared to wild type (*P* < 0.01; Fig. 4d). Interestingly, among those mutant animals, only *unc-22*(*e66*) showed significantly larger bending amplitudes in both dorsal and ventral directions than the wild type (*P* < 0.001; Fig. 4c,d).

**Figure 5:**
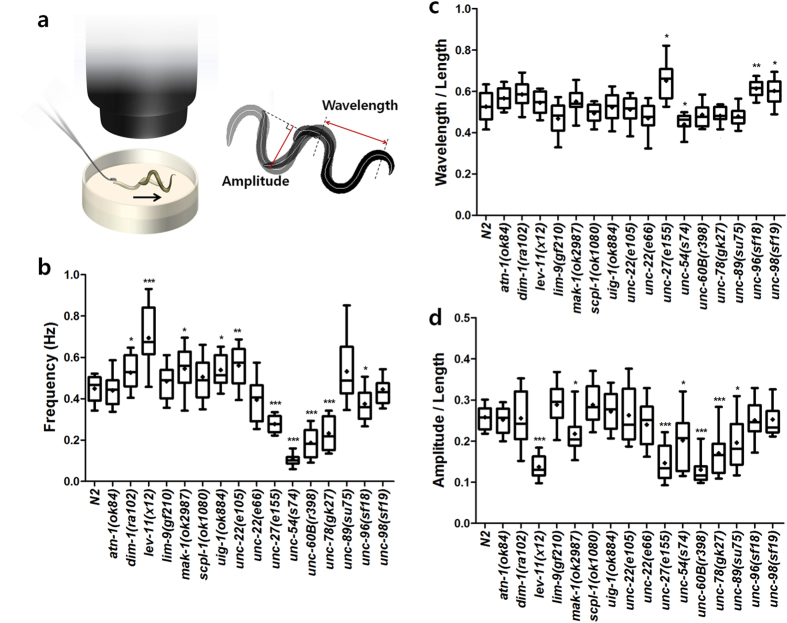
Crawling locomotion analysis of of *C. elegans* sarcomere mutants. **(a)** Schematic diagram of crawling locomotion assays. The nematodes were put on fresh NGM plates solution and their head was gently touched by a platinum wire to induce the backward crawling locomotion. Three quantitative parameters were extracted from the threshold-segmented images: **(b)** frequency, **(c)** wavelength, and **(d)** amplitude of the crawling waves normalized by the length of the animals. *n* ≥ 20. ^*^P < 0.01; ^**^P < 0.001; ^***^P < 0.0001.

For the analysis of crawling behavior, the nematodes were put on the agar surface of a fresh Nematode Growth Medium (NGM) plate and their head was touched with a platinum wire in a gentle manner, leading to their backward crawling motion (Fig. 5a)^16^. From the segmented images, we could extract three features for describing the crawling locomotion: frequency, wavelength, and the maximum amplitude of the crawling waves. According to the results, the crawling frequency of *dim-1*(*ra102*), *lev-11*(*x12*), *mak-1*(*ok2987*), *uig-1*(*ok884*), and *unc-22*(*e105*) was significantly higher than that of the wild type animal, while that of *unc-27*(*e155*), *unc-54*(*s74*), *unc-60B*(*r398*), *unc-78*(*gk27*), and *unc-96*(*sf18*) was significantly lower (*P* < 0.01) (Fig. 5b). The wavelength in crawling waves of *unc-27*(*e155*), *unc-96*(*sf18*), and *unc-98*(*sf19*) was significantly larger than that of the wild type, while that of *unc-54*(*s74*) was significantly smaller than that of the wild type (*P* < 0.01) (Fig. 5c). The amplitude of *lev-11*(*x12*), *mak-1*(*ok2987*), *unc-27*(*e155*), *unc-54*(*s74*), *unc-60B*(*r398*), *unc-78*(*gk27*), and *unc-89*(*su75*) was significantly smaller as compared to that of the wild type (*P* < 0.01) (Fig. 5d).

**Figure 6:**
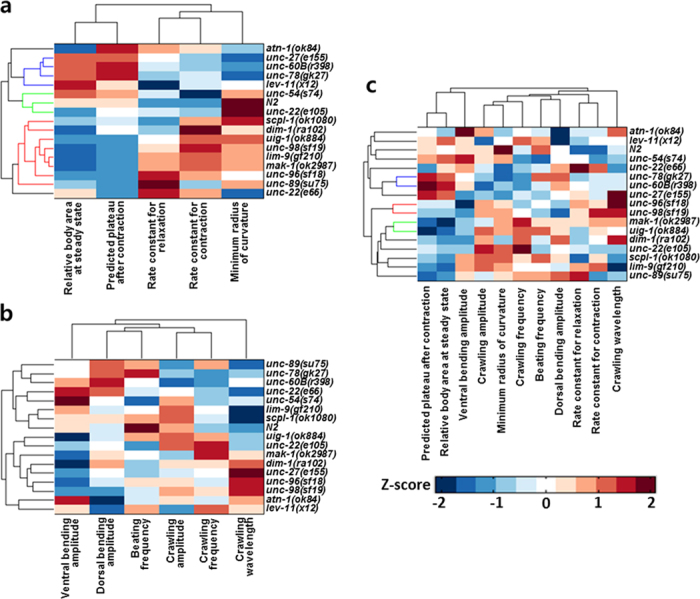
Standard hierarchical analysis based on behavioral phenotypes of *C. elegans* sarcomere mutants. Clustering results of the wild-type and sixteen sarcomere mutant strains based on the Z-score of the mean values of the phenotypic profiles obtained from **(a)** the optogenetic muscle assays, **(b)** the conventional swimming and crawling locomotion assays, and **(c)** both assays. Only the phenotypic analysis enabled by the optogenetic muscle assays provides reliable functional relationships among the sarcomere mutants.

To test whether the features obtained from the optogenetic muscle contraction assays could provide reliable phenotypic profiles to relate to gene function, we conducted a hierarchical clustering analysis based on mean values of the parameters that were extracted from the experiments. From the analysis using the features extracted from the optogenetic muscle assays, the clusters were separated into three groups (Fig. 6a). The mutants having defects in the proteins localized at thin filaments^23^, including *unc-27*(*e155*), *unc-60B*(*r398*), *unc-78*(*gk27*), and *lev-11*(*x12*), were clustered in one group (indicated by blue lines to the right of Fig. 6a). The mutants having defects in proteins localized at M-lines and dense bodies, such as *dim-1*(*ra102*), *lim-9*(*gk210*), *mak-1*(*ok2987*), *scpl-1*(*ok1080*), *uig-1*(*ok884*), *unc-89*(*su75*), *unc-96*(*sf18*), and *unc-98*(*sf19*), are in one cluster (indicated by red lines to the right of Fig. 6a). Interestingly, *unc-22*(*e105*) and *unc-54*(*s74*), which have a normal or nearly normal sarcomere structure^2^,^24^, were clustered in the same group with wild-type animals (indicated by green lines in Fig. 6a), while the mutants having structural sarcomere defects, such as *dim-1*(*ra102*), *lim-9*(*gk210*), *uig-1*(*ok884*), *unc-89*(*su75*), *unc-96*(*sf18*), and *unc-98*(*sf19*), were included in one cluster. While the clustering analysis based on the parameters from the optogenetic assays showed a result that is consistent with current knowledge, we did not find any reliable clusters from the analysis based on the features extracted from the conventional locomotion assays (Fig. 6b). Furthermore, combining the results from the optogenetic assays and those from the conventional locomotion assays also did not result in meaningful clusters (Fig. 6c). This may be due to the ability of the mutant animals to compensate the sarcomere defects during locomotion by changing the kinematics of their locomotion pattern.

## Discussion

Our results demonstrate the feasibility of a new quantitative assay to study the functions of genes encoding sarcomere proteins in striated body wall muscles of *C. elegans,* both as individual genes, and their relationships to each other. Although there are studies using locomotion phenotypes of *C. elegans* muscle mutants based on their swimming or crawling motion^16^,^17^, it has been difficult to study the kinetics of muscle contraction and relaxation processes in these microscopic freely-moving nematodes due to the lack of appropriate tools. Optogenetics, which enables optical control of neuronal activity, has been applied for analyzing neuromuscular synaptic functions^20^,^21^,^22^. Here we applied optogenetics as a tool for studying contraction and relaxation kinetics of the body wall muscles in *C. elegans*: we generated sixteen sarcomere mutant strains carrying ChR2 in their cholinergic motor neurons; a microfluidic device, in which multiple nematodes can be trapped and simultaneously illuminated, and computer vision technology were utilized for high-throughput, non-biased quantification of the body size change due to the light-activated muscle contraction and relaxation processes. It is worth noting that the use of the microfluidic devices greatly enhances the throughput (by reducing the time to locate and identify animals) and the reproducibility (by enhancing the ease of image processing).

We have measured, for the first time, several key parameters of the kinetics of the muscle contraction relaxation cycle, including rate constants for contraction and for relaxation. These data were obtained by studying the contraction/relaxation of the whole animal, the only now practical way such data could be obtained since isolation and manipulation of nematode muscle cells is not currently achievable. We have surveyed a set of 16 muscle-affecting mutant strains (in 15 genes), and found that many are defective in these and related parameters. *unc-54*(*s74*) was the only mutant that showed a decrease in the rate of contraction. This result is perhaps expected. *unc-54*(*s74*) is a missense mutation (Arg to Cys) in the myosin head domain of the major myosin heavy chain of body wall muscle (MHC B), and results in worms with a slow and stiff locomotion, but normal muscle structure^2^,^25^,^26^. As the affected residue lies near the ATP-binding site, it is likely to affect ATP interactions and result in reduced ATPase and motor velocity, and consequently a slower contraction/relaxation cycle of the muscle. It seems significant that none of the 16 mutants tested showed a decrease in relaxation rate. This may be due to muscle relaxation being a passive, not active process. Although in vertebrate muscle elastic recoil from the giant protein titin is involved, a clear titin homolog in nematode muscle does not exist^27^. Of the 16 mutants assayed, 6 showed an increased contraction rate, and 9 showed an increased relaxation rate. Five (*lim-9*, *mak-1*, *unc-22*(*e66*)*, unc-96*, and *unc-98*) showed both increases in contraction and relaxation rates. This suggests that multiple sarcomeric proteins normally inhibit the rates of contraction and relaxation.

Two mutants that showed increased relaxation rates might be explained from what is known or suspected about their roles in muscle activity. *unc-27* encodes one of four troponin I isoforms in *C. elegans*^11^, and at least in vertebrate striated muscle is well known to be involved in inhibiting the interaction of myosin heads with thin filaments^28^. Both mutant alleles of *unc-22*, *e105* and *e66*, showed increased relaxation rates. In fact, *unc-22*(*e66*), yielded the greatest increase in relaxation rate among all 9 mutants that increased relaxation rates. *unc-22* encodes the giant polypeptide twitchin^5^. Both the loss of function “twitching” phenotype, and the presence of a protein kinase domain homologous to the kinase domain of myosin light chain kinase suggested a role for twitchin in regulation of muscle contraction. We suspect that, in fact, *C. elegans* twitchin inhibits the rate of relaxation: (*i*) physiological studies in *Aplysia* suggest that twitchin inhibits the rate of muscle relaxation^29^; (*ii*) in molluscs, some smooth muscles display the “catch” state – a state in which high tension is maintained over long periods with little expenditure of ATP; the release of catch is correlated with protein kinase A phosphorylation of twitchin^30^; and (*iii*) for molluscan muscle, twitchin can be shown to act as a physical linker between thick and thin filaments *in vitro*^31^. It is perhaps significant that the rate constants for relaxation are increased in both alleles of *unc-22*, *e66* which has disorganized sarcomeres, and *e105* which has normally organized sarcomeres^24^. This suggests that at least one function of twitchin is indeed regulatory rather than structural.

We also conducted a clustering analysis to find functional relationships among the genes encoding the sarcomere proteins. Standard hierarchical clustering based on parameters extracted from the optogenetic assays (relative body area at steady state, plateau after contraction, rate constants for relaxation, rate constants for contraction, minimum radius of curvature) yielded clusters of gene/proteins that corroborate with what is known about the functions of these genes/proteins in the sarcomere. One cluster included all the thin filament proteins in our set of 15 proteins (UNC-27(troponin I), LEV-11(tropomyosin), UNC-60B(ADF/cofilin), UNC-78(AIP1)). Another cluster consisted of 8 of 9 of the proteins that are localized to M-lines and/or dense bodies (MAK-1, UNC-89, UNC-98, UNC-96, SCPL-1, DIM-1, UIG-1 and LIM-9). Based on this experience, we suggest that when a new sarcomere component is identified, it would be useful to conduct a similar optogenetic and clustering analysis to provide evidence for how such a new component works together with other known components of the sarcomere. A further indication that the optogenetic approach is powerful is that cluster analysis, using parameters extracted from the conventional locomotion assays, did not reveal any statistically significant clusters. In conventional assays, movement relies on the simultaneously-coordinated contraction of some muscles and relaxation of others. With optogenetic regulation of muscle contractility, we were able to isolate the different phases of contraction and relaxation. Optogenetics enabled us to induce the contraction of body wall muscles to maximum capacity directly, thus revealing aspects of the functions of individual sarcomeric proteins that would be too subtle to be detected by conventional locomotion assays.

## Materials and Methods

### Strains and maintenance

Nematodes were grown at 20 °C on standard nematode growth medium (NGM) plates seeded with *E.coli* OP50 bacteria as a food source. For optogenetic experiments, all-*trans* retinal (Sigma-Aldrich, St. Louis, MO, USA), as an essential cofactor for ChR2 activation, was added to *E. coli* OP50 cultures to a final concentration of 100 μM and spread onto 5.5-cm NGM plates. The animals were kept in the dark until the assay to avoid unwanted ChR2 photoactivation.

Wild-type nematodes were the N2 strain. Strains used in this study are as follows: N2: wild type (Bristol isolate), ZX460: N2;*zxIs6[punc-17::ChR2*(*H134R*)*::YFP;lin-15*^+^*]V*, GB254: *unc-22*(*e105*)*;zxIs6*, GB255: *unc-22*(*e66*)*;zxIs6*, GB256: *mak-1*(*ok2987*)*;zxIs6*, GB257: *unc-89*(*su75*)*;zxIs6*, GB258: *unc-98*(*sf19*)*;zxIs6*, GB259: *unc-54*(*s74*)*;zxIs6*, GB262: *unc-96*(*sf18*)*;zxIs6*, GB263: *dim-1*(*ra102*)*;zxIs6*, GB261: *atn-1*(*ok84*)*;zxIs6*, GB264: *scpl-1*(*ok1080*)*;zxIs6*, GB265: *uig-1*(*ok884*)*;zxIs6*, GB260: *lim-9*(*ok210*)*;zxIs6*, ON295: *unc-27*(*e155*)*;zxIs6*, ON302: *lev-11*(*x12*)*;zxIs6*, ON303: *unc-60B*(*r398*)*;zxIs6*, and ON304: *unc-78*(*gk27*)*;zxIs6*.

### Optogenetic assays

For on-chip optogenetic assays, two-layer microfluidic devices made of polydimethylsolxane (PDMS; Sylgard 184, Dow-Corning Corp., Midland, MI, USA) were fabricated using standard multi-layer soft lithography. The device has 16 parallel microchannels for trapping worms, and their opening and closing are controlled by two valves in the upper layer (Fig. 1a,b). Each channel is 60 μm wide and has a rectangular cross section, thus fluid flow or small animals are allowed to pass through the channels while the valves are partially closed. The valves were filled with a 58% glycerol solution to match the refractive index of the PDMS resulting in improved image quality. The pressures for the valve actuation and the sample delivery were regulated by off-chip solenoid valves (Series 188, ASCO Valve Inc., Florham Park, NJ, USA).

To trap worms in the microchannels, the valve at the exit of the channels were initially closed. After young adult animals were delivered into the channels by a loading pressure, the valve at the entrance of the channels was closed and the loading pressure was turned off. The animals were illuminated with blue light (450–490 nm; 0.3 mW/mm^2^) for 15 s to induce ChR2 photoactivation. For measurements of projected body area, movies were recorded using a CCD camera (Infinity 3-1, Luminera Corp., Canada). After completing the light illumination and image acquisition, the exit valve was open and the loading pressure was turned on to flush out the worms, followed by another cycle. All the processes including valve actuation, worm injection, and image acquisition were controlled by LabVIEW software.

The movies were post-processed using custom software written in MATLAB. Images of the channels without worms were used for removing background of the captured movies, resulting in high quality segmentation of worm images. The projected body area of the worms in the segmented images was used as a read-out for the light-stimulated muscle contraction and relaxation. The body area measured from the images captured during 5 s before the blue light illumination was used as a baseline, and its change was plotted over time. The curves were fitted with the plateau followed by one phase decay or association equations for the kinetic analyses of the contraction or the relaxation processes, respectively.

For on-plate optogenetic assays looking at maximum body bend, young adult animals were transferred to fresh 5.5-cm NGM plates and images were taken to measure their body length with no illumination. The animals were illuminated with blue light until they fully contract their body, then images were taken to measure the minimum radius of curvature of the worm body at full contraction. The measured values were normalized with the length of each animal.

### Locomotion analysis

For swimming or backward locomotion analyses, young adult animals were transferred into 3 cm diameter plates containing 500 μL of M9 buffer or fresh 5.5-cm NGM plates, respectively. After a 2-min acclimation period, the behavior of each animal was observed. To induce backward motion, their head was gently prodded with a platinum wire. For both experiments, movies were acquired on a standard transmitted light stereo microscope.

The movies were post-processed to extract the worm skeleton using custom software written in MATLAB. The average frequencies for swimming or crawling locomotion were measured. The maximum ventral or the dorsal bending amplitudes, or the maximum bending amplitude and the wavelength were measured from the swimming or the crawling locomotion. Those values were normalized by the length of each worm.

### Statistical analysis

All curve fitting and statistical analysis were performed using Prism 5 (GraphPad Software, San Diego, CA, USA). Wilcoxon Rank sum test was conducted for comparison of the data when appropriate. *P*-values < 0.01 were considered as statistically significant: ^*^P < 0.01; ^**^P < 0.001; ^***^P < 0.0001.

### Clustering analysis

A standard hierarchical clustering analysis was conducted using MATLAB. After normalizing the mean values of the parameters obtained from the experiments into Z-score, the phenotypic distances between the strains were calculated. Based on the distance information, the strains were linked, and the hierarchical cluster tree was created.

## Additional Information

**How to cite this article**: Hwang, H. *et al.* Muscle contraction phenotypic analysis enabled by optogenetics reveals functional relationships of sarcomere components in *Caenorhabditis elegans*. *Sci. Rep.* **6**, 19900; doi: 10.1038/srep19900 (2016).

## Supplementary Material

Supplementary Information

## Figures and Tables

**Table 1:** List of fifteen genes encoding sarcomere proteins examined in this study.

Gene	Allele	Type of allele	Protein	Location of protein
*unc-22*	*e66*	cannonical	twitchin	A-bands
*unc-22*	*e105*	missense, 7^th^ Ig domain, GtoR	twitchin	A-bands
*mak-1*	*ok2987*	deletion, null	MAK-1 (MAPKAP kinase 2)	between & around dense bodies
*unc-54*	*s74*	missense, myosin head	MHC B (myosin heavy chain B)	thick filaments
*unc-89*	*su75*	lacks expression of all large isoforms	UNC-89 (obscurin)	M-lines
*unc-98*	*sf19*	splice site, null?	UNC-98 (C2H2 Zn fingers)	M-lines (dense bodies & nuclei)
*unc-96*	*sf18*	nonsense, null	UNC-96	M-lines (& dense bodies)
*unc-27*	*e155*	null	troponin I	thin filaments
*lev-11*	*x12*	missense, E234K	tropomyosin	thin filaments
*unc-60B*	*r398*	lacking 3 aa at C-term	ADF/cofilin	thin filaments
*unc-78*	*gk27*	deletion, null	actin-interacting protein I (AIP1)	thin filaments
*atn-1*	*ok84*	deletion, null	α-actinin	dense bodies
*scpl-1*	*ok1080*	deletion, null	SCPL-1 (small CTD-phosphatase)	M-lines & I-bands
*dim-1*	*ra102*	splice site, null	3 Ig domains	between & around dense bodies
*uig-1*	*ok884*	deletion, null	Cdc42 GEF	dense bodies
*lim-9*	*gk210*	deletion, null	Four and a half LIM domains (FHL)	M-lines & I-bands
