# Supplementary material for: Muscle contraction phenotypic analysis enabled by optogenetics reveals functional relationships of sarcomere components in Caenorhabditis elegans
Source: Sci Rep. 2016 Jan 29;6:19900. doi: 10.1038/srep19900 (PMC4731793; doi:10.1038/srep19900)
Supplement: Supplementary Information [file srep19900-s1.pdf]

Supplementary Information

Behavioral phenotypic analysis enabled by optogenetics reveals functional relationships of sarcomere components in *Caenorhabditis elegans*

Hyundoo Hwang[1], Dawn E. Barnes[2], Yohei Matsunaga[2], Guy M. Benian[2], Shoichiro Ono[2], Hang Lu[1,3,4]

[1]School of Chemical & Biomolecular Engineering, Georgia Institute of Technology, Atlanta, GA, USA, [2]Department of Pathology, Emory University, Atlanta, GA, USA, [3]Interdisciplinary Program of Bioengineering, Georgia Institute of Technology, Atlanta, GA, USA, [4]The Petit Institute for Bioengineering and Biosciences, Georgia Institute of Technology, Atlanta, GA, USA.

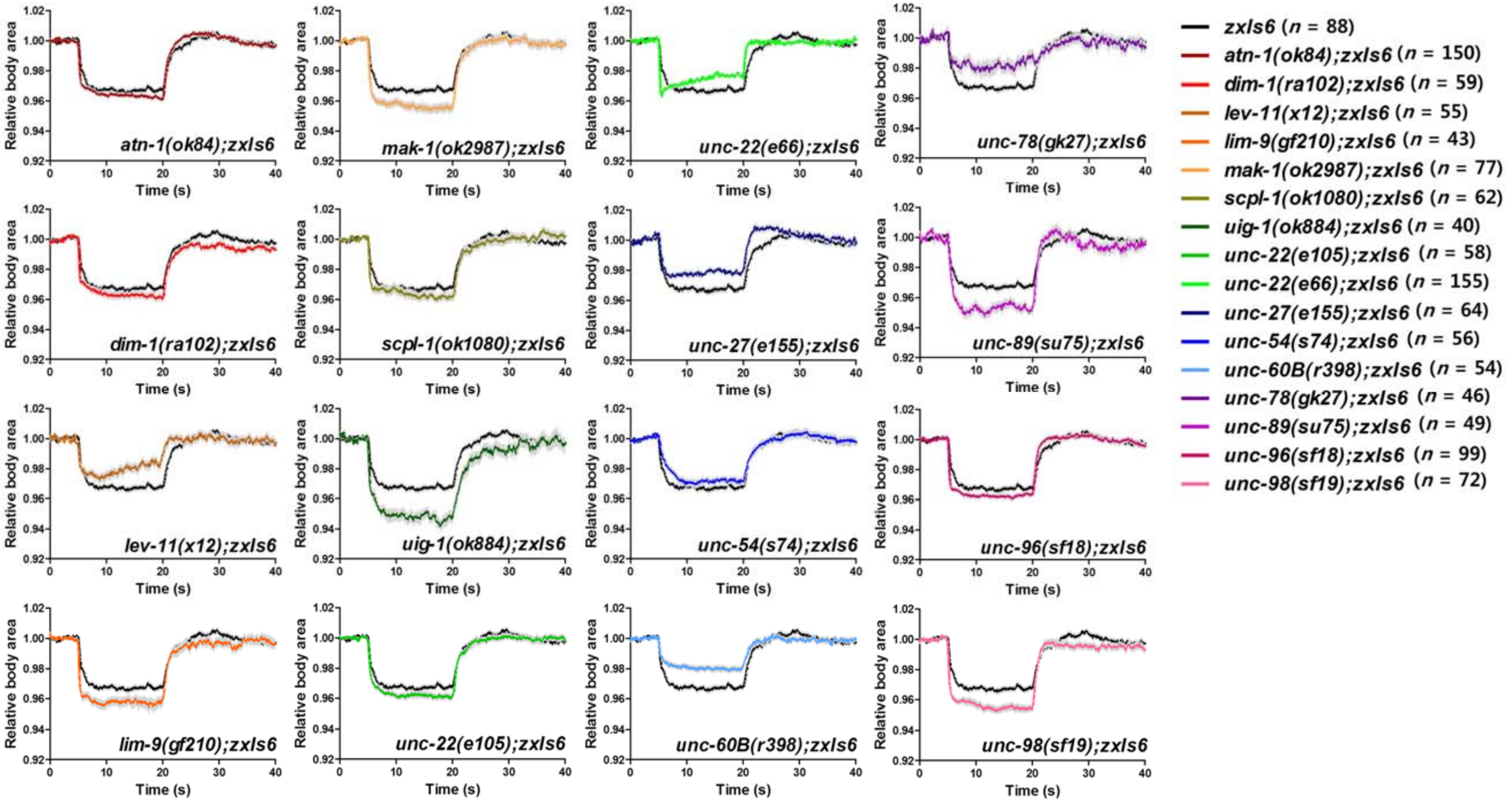

Supplementary Figure 1 | Body size changes of the *C. elegans* sarcomere mutant strains due to the optogenetically-induced muscle contraction. All the data are plotted in comparison with the wild-type (black solid line). Blue light was illuminated 5 s after starting the experiments for 15 s, and the body sizes were normalized by the average values of the first 5 s of each measurement. Data represent mean ± s.e.m. $n \geq 40$.
